# The Potential Clinical Utility of Pressure-Based vs. Heat-Based Paradigms to Measure Conditioned Pain Modulation in Healthy Individuals and Those With Chronic Pain

**DOI:** 10.3389/fpain.2021.784362

**Published:** 2021-12-24

**Authors:** Rima El-Sayed, Camille Fauchon, Junseok A. Kim, Shahrzad Firouzian, Natalie R. Osborne, Ariana Besik, Emily P. Mills, Anuj Bhatia, Karen D. Davis

**Affiliations:** ^1^Institute of Medical Science, University of Toronto, Toronto, ON, Canada; ^2^Krembil Brain Institute, Division of Brain, Imaging, and Behaviour, University Health Network, Toronto, ON, Canada; ^3^Department of Anesthesia and Pain Medicine, Toronto Western Hospital, University of Toronto, Toronto, ON, Canada; ^4^Department of Surgery, University of Toronto, Toronto, ON, Canada

**Keywords:** conditioned pain modulation (CPM), stimulus modality, cuff algometry, heat thermode, chronic pain, antinociception

## Abstract

Conditioned pain modulation (CPM) is a physiological measure thought to reflect an individual's endogenous pain modulation system. CPM varies across individuals and provides insight into chronic pain pathophysiology. There is growing evidence that CPM may help predict individual pain treatment outcome. However, paradigm variabilities and practical issues have impeded widespread clinical adoption of CPM assessment. This study aimed to compare two CPM paradigms in people with chronic pain and healthy individuals. A total of 30 individuals (12 chronic pain, 18 healthy) underwent two CPM paradigms. The heat CPM paradigm acquired pain intensity ratings evoked by a test stimulus (TS) applied before and during the conditioning stimulus (CS). The pressure CPM paradigm acquired continuous pain intensity ratings of a gradually increasing TS, before and during CS. Pain intensity was rated from 0 (no pain) to 100 (worst pain imaginable); Pain50 is the stimulus level for a response rated 50. Heat and pressure CPM were calculated as a change in TS pain intensity ratings at Pain50, where negative CPM scores indicate pain inhibition. We also determined CPM in the pressure paradigm as change in pressure pain detection threshold (PDT). We found that in healthy individuals the CPM effect was significantly more inhibitory using the pressure paradigm than the heat paradigm. The pressure CPM effect was also significantly more inhibitory when based on changes at Pain50 than at PDT. However, in individuals with chronic pain there was no significant difference in pressure CPM compared to heat or PDT CPM. There was no significant correlation between clinical pain measures (painDETECT and Brief Pain Inventory) and paradigm type (heat vs. pressure), although heat-based CPM and painDETECT scores showed a trend. Importantly, the pressure paradigm could be administered in less time than the heat paradigm. Thus, our study indicates that in healthy individuals, interpretation of CPM findings should consider potential modality-dependent effects. However, in individuals with chronic pain, either heat or pressure paradigms can similarly be used to assess CPM. Given the practical advantages of the pressure paradigm (e.g., short test time, ease of use), we propose this approach to be well-suited for clinical adoption.

## Introduction

Conditioned pain modulation (CPM) is a behavioral phenomenon that reflects an individual's inherent capacity to modulate their pain. CPM can be evoked experimentally using “pain inhibits pain” type psychophysical tests (1). Numerous studies have demonstrated the potential clinical utility of CPM to predict the effectiveness of therapeutic approaches that target mechanisms of CPM (2–4).

The CPM effect [a term coined by Yarnitsky et al. (5)] refers to any change in the intensity of pain that is evoked by a test stimulus (TS) applied to one area of the body due to the presence of a concurrent conditioning stimulus (CS) applied to another area of the body (6). This psychophysical measure of CPM designed for testing in humans was motivated by the discovery of the diffuse noxious inhibitory control (DNIC) effect observed in animal electrophysiological single neuronal recordings. Decades of DNIC studies have shown that a noxious stimulus activates a spino-bulbar-spinal feedback loop such that spinal nociceptive projection neurons activate neurons in the brainstem subnucleus reticularis dorsalis (SRD) (7–9). The SRD then activates descending projections through the dorsolateral funiculus, that ultimately inhibits ipsilateral wide dynamic range (WDR) spinal dorsal horn neurons, and thus attenuates their response to a second incoming noxious stimulus (9, 10). However, unlike the inhibitory DNIC effect in animals, the CPM effect in human can be inhibitory or facilitatory. It is now clear that CPM can vary across a wide spectrum, from reduced pain due to the presence of a CS (inhibitory CPM) to increased pain (facilitatory CPM), and in some cases CPM may not occur at all (no-CPM) (6, 11, 12).

Individual factors contribute to the variability of CPM across the population. A systematic meta-analysis in many chronic pain conditions found that on average, people with chronic pain exhibit a weaker inhibitory CPM effect compared to healthy individuals (13). For example, weaker inhibitory CPM has been reported in studies of people with neuropathy, fibromyalgia, irritable bowel syndrome, osteoarthritis, tension-type headache and whiplash-associated disorders (6, 13). Furthermore, there is evidence that an individual's CPM may be used as a clinical measure to guide personalized treatment selection. For example, in a study of people undergoing treatment for painful diabetic neuropathy with the serotonin-noradrenaline reuptake inhibitor (SNRI) duloxetine, patients with weaker inhibitory CPM (thought to reflect a weaker anti-nociceptive pathway) benefited more than those with a stronger inhibitory CPM (3). Furthermore, the improvement in clinical pain was observed alongside an improvement of post-treatment CPM. Thus, this patient-specific treatment outcome was thought to be due to the action of this SNRI to strengthen the descending anti-nociceptive serotonergic and adrenergic neurotransmission that is part of the spino-bulbar-spinal loop. A link between CPM and pain treatment outcome was also found in two studies of osteoarthritis, where patients' CPM shifted to more closely resemble that of the healthy group following a successful knee or hip surgery treatment (14, 15).

Studies of CPM in pain-free individuals are also important not only to glean insight into basic mechanisms of pain modulation, but also to determine its utility in risk assessment for the potential development of chronic pain. For example, compared to the quantitative sensory tests for pain thresholds and suprathreshold pain assessed before a thoracotomy, stronger inhibitory CPM was the only measure that predicted the lower risk of developing chronic post-surgery pain (4). A similar finding was also reported for patients undergoing cesarean and major abdominal surgeries (16, 17). Therefore, assessing CPM has potential clinical utility to predict the risk of persistent post-operative pain, as well as to predict the efficacy of therapeutic approaches that target endogenous pain modulation, which can ultimately guide treatment plans for chronic pain management.

Despite decades of research in the field of DNIC and CPM, there remains challenges to adopting a CPM test for clinical use. Practical issues can be major factors that impact translating CPM testing from an experimental research tool into a clinical tool. Thus, it is important to establish methodology that is easy to administer and conducive to a clinical setting. For example, there have been recent pursuits to establish a new simple pressure pain stimulator that can induce CPM for bed-side testing (18). Additionally it has been suggested that clinical translation of CPM could be helped by increasing clinical experimental data that assesses the dependency of CPM on stimulus test modalities (19). In the past, CPM has been assessed with paradigms that use different types of stimulus modalities (e.g., heat, cold, electrical and pressure) and there are also different metrics used to quantify the CPM effect (e.g., a change in suprathreshold pain ratings vs. pain detection thresholds). The assumption in the field has been that different stimulus modalities produce basically the same CPM effect, however this has not been definitively established. In 2015, the growing need to reduce variability and standardize the CPM paradigm led a group of experts to recommend the use of either heat or pressure stimulus based paradigms (20). However, since that time, the field has continued to evolve without any particular paradigm being established as a gold standard. Therefore, the aim of the current study was to use a within-subject analysis to assess a commonly used heat-based paradigm with a presumptive simpler pressure-based paradigm in healthy individuals and those with chronic pain. We hypothesized that CPM based on a heat vs. a pressure paradigm would not differ significantly in an individual (healthy or with chronic pain).

## Materials and Methods

### Participants

The study consisted of two groups: 1) healthy individuals recruited through advertisements posted throughout the University Health Network, Toronto, Canada and through word of mouth, and 2) people with chronic pain who were recruited as part of a larger, ongoing study of chronic pain. All study participants provided informed consent for the procedures approved by the University Health Network Research Ethics Board. All study participants underwent evaluation of CPM using both a heat pain-based paradigm and a pressure pain-based paradigm, allowing for both within-subject and group evaluations. The CPM data in this study were collected as part of a large battery of psychophysical tests for studies of acute and chronic pain. Healthy participants were excluded if they had 1) current ongoing pain or a history of chronic pain (pain lasting >3 months) 2) any major chronic health condition, or 3) a psychiatric disorder, neurological disorder, or a Beck inventory Depression (BDI) score (range 0–63) >13 (indicating greater than minimal self-reported depression). The chronic pain group consisted of people with chronic pain who were awaiting a spinal cord stimulation trial for pain management due to failed back surgery syndrome with back and/or lower limb pain (*n* = 7), complex regional pain syndrome in the lower limbs (*n* = 3), post-traumatic neuropathic pain in the lower limb (*n* = 1), and occipital neuralgia (*n* = 1).

### Evaluation of Conditioned Pain Modulation

**Figure 1:**
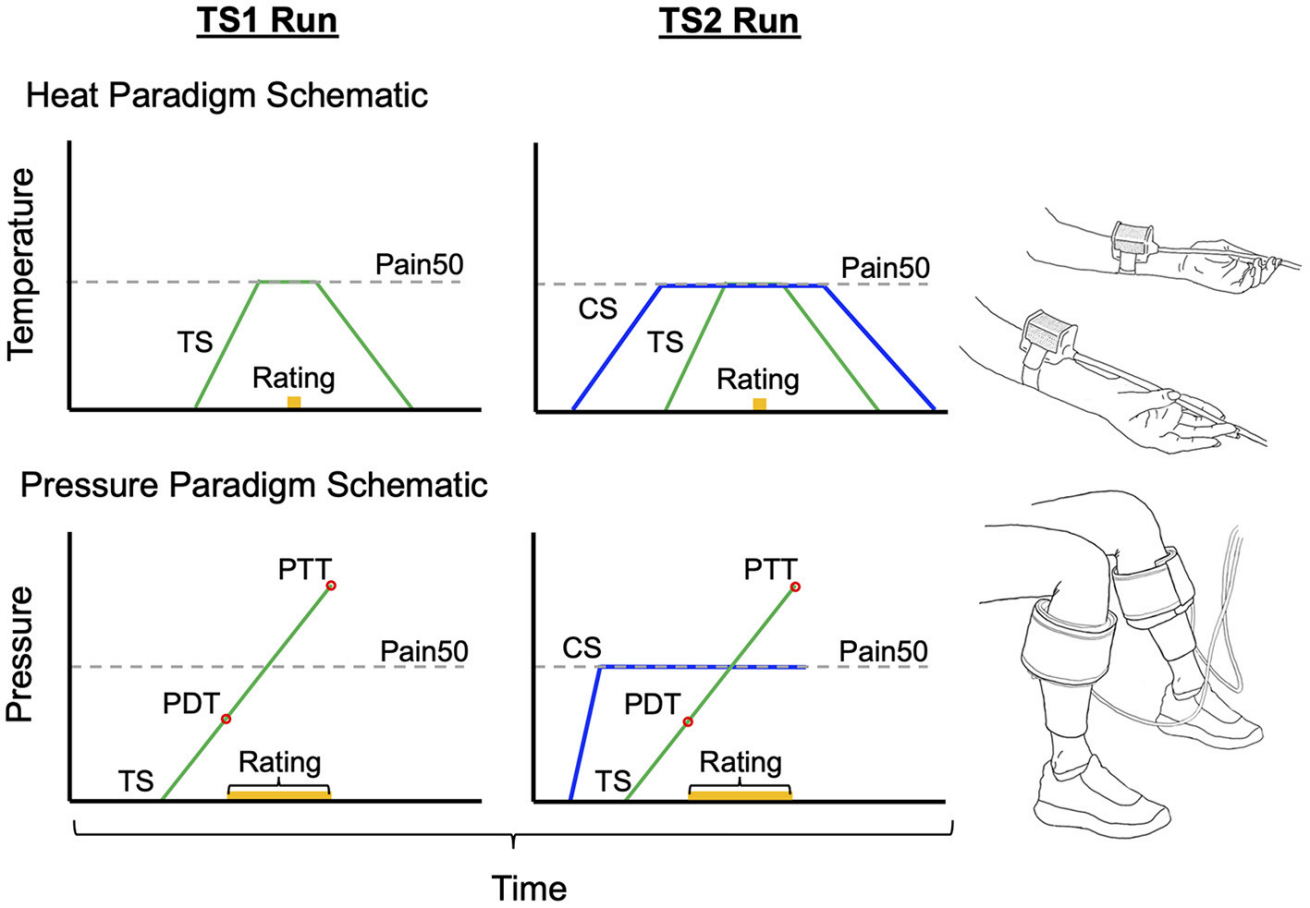
Schematics to represent the stimulus order given in each CPM paradigm and the standard setup. In the heat paradigm the test stimulus (TS) is held at the Pain50 temperature only while the pain rating is obtained. In the pressure paradigm the TS continues to rise after Pain50 until they reach their pain tolerance. However, the pain rating at Pain50 pressure is extracted from TS1 and TS2 in order to calculate CPM similarly to the heat paradigm. Both are parallel sequence paradigms [conditioning stimulus (CS) in blue overlaps with TS2 when the second pain rating is obtained]. Pain50 is the stimulus intensity that evokes a pain rating of 50/100, where 0 is no pain and 100 is the worst pain imaginable. PDT, pain detection threshold; PTT, pain threshold tolerance.

In the heat paradigm, stimuli were delivered to the volar forearms through two 30 × 30 mm contact thermodes (QSense device; Medoc Ltd, Israel) (Figure 1). In the pressure paradigm, stimuli were delivered to the calves through two inflatable 10 × 61 cm pressure cuffs (CPAR, NociTech Inc., Denmark) (Figure 1). In individuals with chronic pain, the cuff was applied to the upper arm bicep if their chronic pain included the leg. This was to ensure that CPM was tested in both paradigms at a body region that was not affected by the chronic pain condition. Stimulus-evoked pain intensity was rated on a scale from 0 to 100 (0 being no pain at all and 100 being the worst pain imaginable) in both paradigms. Participants provided these pain intensity ratings verbally during the heat paradigm and manually using a visual analog scale (VAS) slider during the pressure paradigm.

#### Conditioned Pain Modulation Calculation

The test stimulus (TS) and conditioning stimulus (CS) were set individually for each participant at an intensity that evoked a pain intensity rating of ~50 out of 100 (known as Pain50). The CPM paradigm used was a parallel sequence paradigm where the CS was given concurrently with the second TS as follows: (1) pain intensity is rated during a TS (TS1), (2) a sustained CS is applied to the contralateral body region, (3) during the CS, the pain intensity of the second test stimulus (TS2) is rated. The CPM effect was calculated as a percentage using the following formula:


$$\begin{array}{l} CPM\;Effect\;\%=\frac{TS2\;Pain\;rating-TS1\;Pain\;rating}{TS1\;Pain\;rating}\;\times \;100\% \end{array}$$

Therefore, a negative CPM effect is indicative of inhibitory CPM where a concurrent CS results in a lower pain rating of the second TS. A positive CPM effect is indicative of facilitatory CPM where a concurrent CS results in a higher pain rating of the second TS. Lastly, 0% indicates no CPM effect, where the concurrent CS did not change the pain rating of the TS.

Pain ratings at Pain50 were determined for both heat and pressure paradigms. In the pressure paradigm, in addition to the Pain50 measure used to calculate CPM, we determined the pressure pain detection threshold (PDT) and pain tolerance threshold (PTT) because previous studies have used these metrics to calculate the CPM effect. To be consistent with designating a negative CPM effect as reflecting inhibitory CPM, we calculated CPM from the pressure pain detection threshold (PDT) with the formula:


$$\begin{array}{l} CPM\;Effect\;\%=\frac{TS1\;PDT-TS2\;PDT}{TS1\;PDT}\;\times \;100\% \end{array}$$

#### Heat-Based CPM Paradigm

For each participant, prior to the CPM test, a familiarization paradigm was used to determine their Pain50. In this paradigm, participants rated the pain intensity that was evoked by each of the six heat stimuli in the following order: 44, 45, 43, 46, 42, and 47°C. Since the aim was to find a temperature that evokes a pain intensity rating of 50/100, if any of the first five stimuli evoked a pain intensity rating >75/100, then the last 47°C did not need to be tested. Each of these familiarization test stimuli were delivered from a baseline temperature and interstimulus temperature of 35°C for 15 s and a ramp-up rate of 2°C/s to reach the target temperature which was held for 6 s. After the temperature was at the target temperature for 3 s, participants were prompted to rate the evoked pain intensity and the thermode temperature returned to baseline at a rate of 1°C/s. The temperature that evoked Pain50 was estimated from the familiarization paradigm. We then confirmed that this stimulus did evoke a pain rating of 50/100 during several TS that were part of a habituation paradigm (TS had identical timing and ramp rates to the TS in the CPM paradigm below). The Pain50 TS and CS temperatures were then manually set based on the result of the familiarization and habituation paradigm.

To test CPM, one thermode delivered the TS at 2°C/s from a 35°C baseline to the target Pain50 temperature. The temperature was held at this target for 7 s at which point the participant verbally provided a rating of their pain intensity, and then the temperature decreased back to baseline at 1°C/s (Figure 1). The second thermode delivered the CS to the contralateral forearm, the temperature increased from baseline at 1°C/s to the Pain50, was held there for 100 s, and then returned to baseline at 1°C/s (Figure 1). The TS1 started after 5 s of baseline and the TS2 was delivered 69 s after TS1 (i.e., during the CS). The full heat-based protocol (familiarization, habituation, and CPM) required ~16 min to complete.

#### Pressure-Based CPM Paradigm

In the pressure paradigm, pain was rated continuously from the moment the TS starts to feel painful (i.e., the threshold for detecting pressure pain, PDT) until the tolerance level is reached (i.e., the threshold for pain tolerance, PTT). Previous studies using pressure paradigms commonly calculate the CPM effect as a change in PDT between TS1 and TS2 (21–23). The PDT is recorded when the VAS slider is moved from 0 to 0.1 cm. In addition to evaluating CPM using PDT, we evaluated CPM effect as a change in pain rating at Pain50 between TS1 and TS2 (similarly to the heat paradigm). To determine these pain ratings from the continuous pain ratings, first we found the initial pressure during TS1 that evoked a pain intensity rating of 50/100 (Pain50, indicated when the slider was at 5.0 cm along the 10 cm length). Then the TS2 pain rating used to calculate the CPM effect was the pain intensity rated when the TS2 pressure was at Pain50.

In each participant, prior to the CPM test, a threshold paradigm was used three times (separated by 1 min) for familiarization, determining the CS pressure, and to deliver the TS1 (Figure 1). In each trial, the pressure in one cuff continuously increased at a rate of 1 kPa/s and the participant used a VAS slider to continuously rate the evoked pain intensity. The VAS slider scale was labeled with words and numbers; “No pain” at 0 and “Most intense pain imaginable” at 100. The participants were instructed to press a button on the slider when they reached their PTT; pressing this button then deflated the cuff. The maximum cuff pressure allowable was 100 kPa, with the cuff automatically deflating if it reached this level. The first trial on the right limb was only used to familiarize the participant with the protocol. For the second trial, the stimulus was delivered to the opposite limb. The software for the pressure system set the CS pressure level for the CPM test at 70% of the PTT from the second trial. The third trial on the right limb was the TS1.

The CPM assessment consisted of determining the TS2 pain on the right limb in the presence of a concurrent CS delivered to the opposite limb (Figure 1). To do this, the CS rapidly increases to the set pressure and is held at that level for 100 s. At the same time the TS (TS2) pressure gradually increased with the same protocol given as the TS1 test (participants continuously rated the pain intensity evoked by the TS until the TS reached their PTT where they then press the button). Pressing the button deflates both cuffs. This paradigm takes ~10 min.

### Statistical Analyses

All correlation and group statistical analyses were performed using R (version 3.6.0; https://www.r-project.org) in RStudio (version 1.0.44; https://www.rstudio.com). GraphPad Prism (version 7.03, https://www.graphpad.com) was used to create the figures and Microsoft Excel (version 2010; microsoft.com/excel) was used for some descriptive statistics (mean and standard deviation). The Shapiro-Wilk test was used to assess the normality of the data distribution that is required to subsequently run a parametric 2-tailed test. If the distribution passed the normality test a paired *t*-test (*t* statistic) was used to evaluate within-subject differences, otherwise the Wilcoxon signed rank test (*W* statistic) was used. For the paired *t*-test analyses the common measure of effect size Cohen's *d* is reported which had the same conclusion (small, medium, or large effect size) when assessed using Hedges' g (24). The effect size for the Wilcoxon signed rank test analyses is *r* (*z* statistic divided by the square root of the sample size) (25). For between group comparisons if both groups were normally distributed, an independent *t*-test (welch two-sample *t*-test in *R, t* statistic) was used otherwise the Mann-Whitney Wilcoxon test (*W* statistic) was used. The comparison of sex difference proportions between the healthy and chronic pain group was assessed using two-proportions *z*-test (*X*^2^ statistic). Data passed the Shapiro-Wilk normality test before being correlated using the Pearson correlation test (*r* statistic). In the results section, bracketed values followed by a ± symbol represent the mean ± the standard deviation.

## Results

### Demographics and Descriptive Statistics

Data were collected from a total of 30 participants (18 healthy controls, 12 people with chronic pain). There were no significant differences in the proportion of females and males across the healthy group (9F, 9M) and the chronic pain group (7F, 5M) (*X*^2^ = 0.006, *p* > 0.05). However, there was a significant difference in mean age across the chronic pain group (55.3 ± 15.6 years old) and healthy control group (31.8 ± 11.0 years old; *W* = 26, *p* < 0.01). The average BDI scores were also significantly higher (*W* = 39, *p* < 0.01) in the chronic pain group (9.7 ± 5.1) compared to the healthy group (3.9 ± 4.0).

Of the three CPM measures, the heat CPM and the PDT pressure CPM were not significantly different between the healthy and chronic pain group (*p* < 0.05). However, the pressure CPM (at Pain50) was significantly more inhibitory in the healthy group compared to the chronic pain group (*t* = −2.23, *p* = 0.04). Additional descriptive statistics for each CPM paradigm and group can be found in Table 1. The following result sections highlight within-subject comparisons.

**Table 1:** Group demographics and CPM descriptive statistics.

**Variable**	**Healthy group**	**Chronic pain group**
*N* (F, M)	18 (9, 9)	12 (7, 5)
Age (Y)	31.8 ± 11.0^*^	55.3 ±15.6^*^
BDI	3.9 ± 4.0^*^	9.7 ± 5.1^*^
PDT Pressure CPM Effect (% change)	−14.6 ± 32.4	−26.6 ± 48.7
PDT Pressure CPM Effect (absolute change)	−3.1 ± 6.6	−3.6 ± 9.3
TS1 pressure (kPa)	22.3 ± 9.7	22.7 ± 9.0
TS2 pressure (kPa)	25.4 ± 13.4	26.3 ± 8.8
Pain50 Pressure CPM effect (% change)	−50.1 ± 33.0^*^	−21.4 ± 35.8^*^
Pain50 Pressure CPM effect (absolute change)	−25.2 ± 16.6	−10.8 ± 17.9
TS1 pressure pain rating (0–100)	50.3 ± 0.4	50.2 ± 0.4
TS2 pressure pain rating (0–100)	25.1 ± 16.5	39.4 ± 17.8
TS Pain50 pressure (kPa)	36.9 ± 14.1	46.8 ± 15.0
CS pressure (70% PTT) (kPa)	33.7 ± 12.5	50.3 ± 12.9
CS pressure Pain50 (kPa)	34.2 ± 13.4	47.9 ± 14.4
Pain50 Heat CPM effect (% change)	−6.5 ± 32.3	−11.1 ± 33.6
Pain50 Heat CPM effect (absolute change)	−3.6 ± 16.4	−6.5 ± 16.4
TS1 heat pain rating (0–100)	50.5 ± 9.3	49.6 ± 16.3
TS2 heat pain rating (0–100)	46.9 ± 18.5	43.1 ±17.9
TS Pain50 temperature (°C)	45.7 ± 1.4	44.6 ± 3.0
CS Pain50 temperature (°C)	45.4 ± 1.4	44.3 ± 2.4
painDetect score (NNP, MNP, NP)	NA	19.7 (2, 2, 8) ± 8.5
BPI Pain Severity score	NA	6.3 ± 1.0
BPI Interference score	NA	6.0 ± 1.8

*Group data are shown as mean ± standard deviation. N, Number of participants; F, Female; M, Male; BDI, Beck Depression Inventory; PDT, Pain Detection Threshold; TS1, first test stimulus; TS2, second test stimulus; CS, conditioning stimulus; Pain50, stimulus evoking pain rating of 50/100; CPM, conditioned pain modulation; PTT, pain tolerance threshold; NP, Neuropathic Pain; MNP, Mixed-NP; NNP, non-NP; BPI, Brief Pain Inventory. Note that the CS pressure used during pressure-based CPM was at 70% PTT. Asterisks denote statistically significant difference between healthy and chronic pain group (p < 0.05)*.

### Relationship Between CPM Effect and Stimulus Modality

The TS pain ratings during the heat paradigm were only collected at Pain50. Therefore, the following comparisons of CPM between the heat and pressure paradigm are all from CPM calculated as a change in TS pain ratings at Pain50; with inhibitory CPM being a negative % and facilitatory CPM being a positive %.

**Figure 2:**
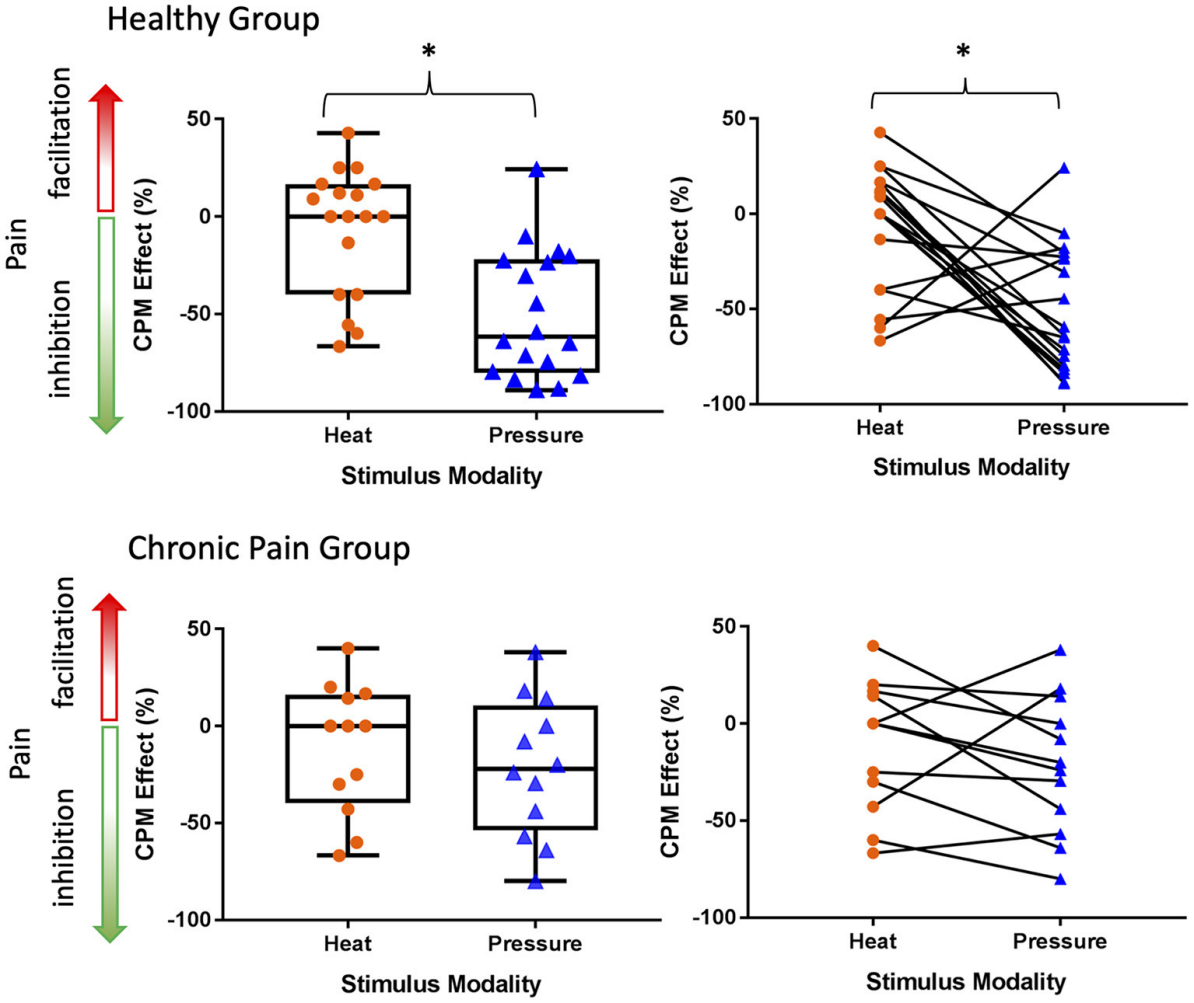
The CPM effect only significantly differs between stimulus modalities in the healthy group. In both the healthy group and chronic pain group the inhibitory CPM effect is more pronounced using pressure than heat stimuli. Line within box is the median, edges of box are 25th to 75th percentiles, and the whiskers denote the maximum and minimum. Asterisks denote significant difference within-subjects (*p* < 0.05).

Overall, in the healthy individuals, the CPM effect was significantly different between heat and pressure paradigms (*t*-test: *t* = −3.41, *p* = 0.004, Cohen's *d* = −1.34; see Figure 2); where the CPM effect in the pressure paradigm (−50.1 ± 33.0%) is on average more inhibitory compared to the heat paradigm (−6.5 ± 32.3%). In the chronic pain group, the CPM effect in the pressure paradigm on average was more inhibitory (−21.4 ± 35.8%) than the heat paradigm (−11.1 ± 33.6%), but there was no significant difference between modalities at the individual level (*t*-test: *t* = −1.05, *p* = 0.32, Cohen's *d* = −0.30; Figure 2).

**Figure 3:**
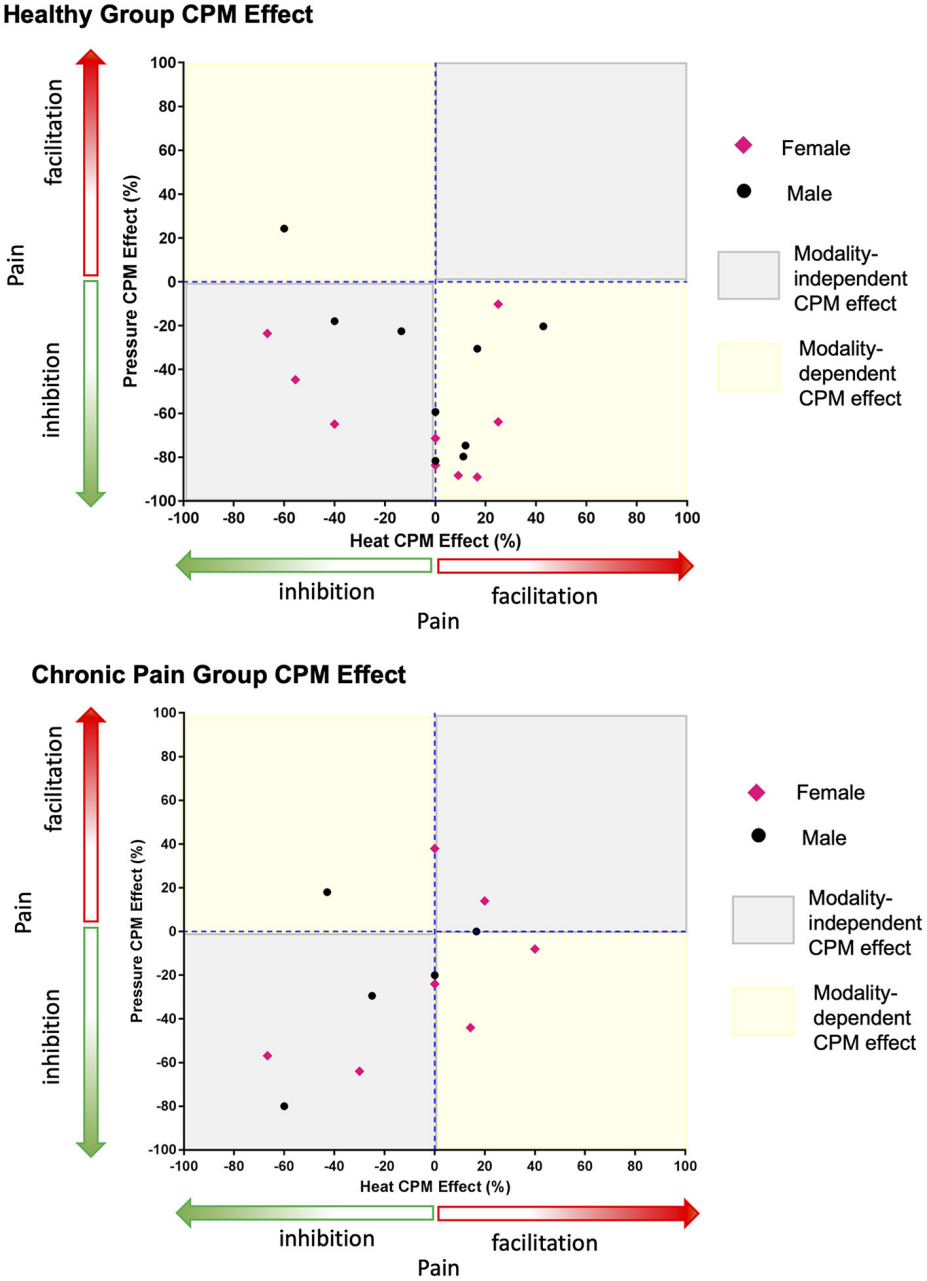
Greater number of healthy individuals demonstrate a modality-dependent CPM effect compared to individuals with chronic pain. Points that fall in the modality-dependent yellow quadrants reflect individuals who could be classified on the opposite ends of the CPM effect spectrum (facilitatory vs. inhibitory) depending on whether the paradigm is heat or pressure based. Modality-independent gray quadrants reflect individuals who would have the same type of CPM effect regardless of the paradigm.

The within-individual data plots in Figure 2 reveal that subjects either exhibited the same (i.e., modality independent) or opposite (i.e., modality dependent) type of CPM effect in the heat and pressure paradigms. Overall, CPM in most of the healthy individuals was modality-dependent but most of the individuals with chronic pain had modality-independent CPM effects (Figure 3). In the healthy group, only five individuals exhibited modality-independent CPM (inhibitory CPM effect regardless of paradigm). However, modality-dependent CPM effects were found for nine individuals: eight had exhibited a facilitatory heat CPM effect and an inhibitory pressure CPM effect while one had an inhibitory heat CPM effect and a facilitatory pressure CPM effect. Four individuals did not exhibit CPM from the heat paradigm but had an inhibitory CPM effect from the pressure paradigm. There was no clear modality-dependent pattern for healthy males and females (Figure 3).

In the chronic pain group, five individuals had modality-independent CPM effect: four with inhibitory and one with facilitatory CPM effects. Amongst the chronic pain group, three individuals had a modality-dependent CPM effect: two with facilitatory heat CPM and inhibitory pressure CPM, while one had inhibitory heat CPM and facilitatory pressure CPM. Three individuals exhibited no heat CPM, of which two had inhibitory pressure CPM and one had facilitatory pressure CPM. One individual with chronic pain exhibited no pressure CPM with facilitatory heat CPM. There was no clear modality-dependent pattern for males and females with chronic pain (Figure 3).

### Pressure CPM Calculated Using Pain50 Pain Ratings vs. PDT

**Figure 4:**
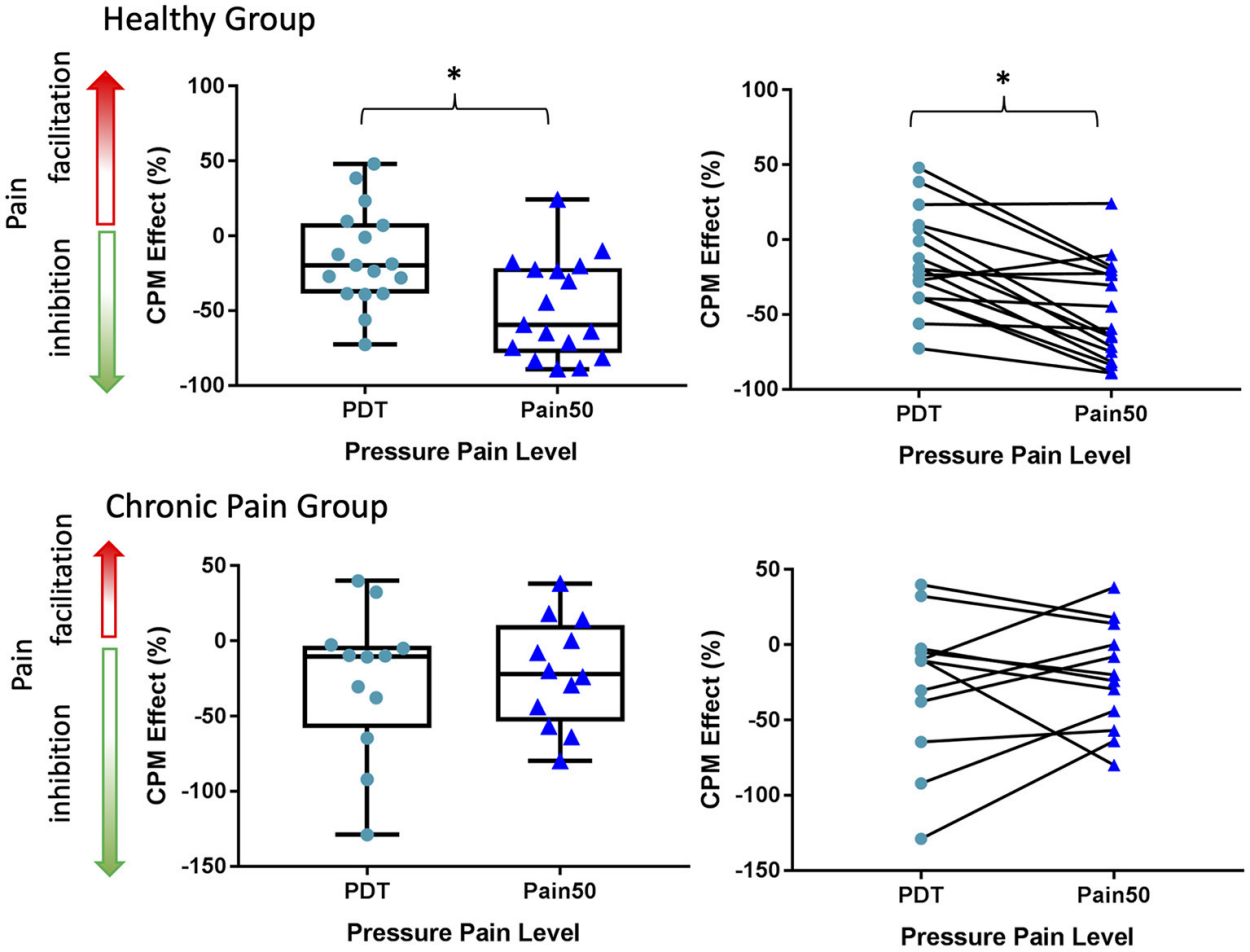
A comparison of CPM effects based on changes in Pain50 vs. the PDT. The CPM effect at PDT and Pain50 only significantly differs in the healthy group. In both the healthy and chronic pain group the inhibitory CPM effect is more pronounced when calculated as a change in pain ratings at Pain50 than a change in the pressure pain detection threshold (PDT). Pain50 is the stimulus intensity that evokes a pain rating of 50/100, where 0 is no pain and 100 is the worst pain imaginable. Line within box is the median, edges of box are 25th to 75th percentiles, and the whiskers denote the maximum and minimum. Asterisks denote significant difference within-subjects (*p* < 0.05).

A within-subject analysis was used to assess the difference in CPM effects based on a change in pressure PDT vs. TS pain intensity ratings at Pain50 (Figure 4). One healthy participant was deemed to be an outlier in terms of their PDT and was excluded from this analysis because they had an extremely low TS1 PDT that was not consistent with other participants or with their own pain thresholds responses from other trials during their psychophysical testing, and thus likely was due to attentional or other effects. Within the healthy participant group, the CPM effect was significantly different between these two measures of CPM (*t* = −4.76, *p* = 0.0002, Cohen's *d* = −1.03). Specifically, the healthy participants exhibited a significantly more inhibitory CPM effect (−48.4 ± 33.1%) when measured as Pain50 CPM compared to PDT CPM (−14.6 ± 32.4%). In contrast, in the chronic pain individuals there was no significant difference (*t* = 0.46, *p* = 0.66, Cohen's *d* = 0.12) in the Pain50 CPM (−21.4, ± 35.8%) compared to the PDT CPM (−26.6 ± 48.7%).

### Difference Between CS Pressure at Pain50 and 70% PTT

The standard software that drives the NociTech Inc. pressure cuff system sets the CS pressure at 70% PTT for the CPM test. While we compared the CPM heat and pressure paradigms with TS pain intensity ratings at Pain50, the CS in the heat paradigm was set to Pain50 while in the pressure paradigm the CS was set to 70% PTT. Therefore, this analysis assesses whether the pressure at Pain50 was significantly different from the 70% PTT used to set the CS.

The healthy group data was not normally distributed (*W* = 0.87, *p* = 0.02) and so was assessed using non-parametric statistics. This indicated that there was no significant difference (Wilcoxon signed rank test: *V* = 105, *p* = 0.42, *r* effect size = 0.20) between the CS pressure at Pain50 (34.2 ± 13.4 kPa, median = 35.9 kPa) and the CS pressure at 70% PTT (33.7 ± 12.5 kPa, median = 34.6 kPa). Similarly, in the chronic pain group, there was no significant difference (*t* = −0.68, *p* = 0.51, Cohen's *d* = −0.18) between the CS pressure at Pain50 (47.9 ± 14.4 kPa, median = 49.6 kPa) and the CS pressure at 70% PTT (50.3 ± 12.9 kPa, median = 52.7 kPa). For comparisons purposes this was also checked using the non-parametric test, and a similar conclusion was found using the Wilcoxon signed rank test (*V* = 35, *p* = 0.79).

### Relationship Between CPM and Clinical Pain Parameters

**Figure 5:**
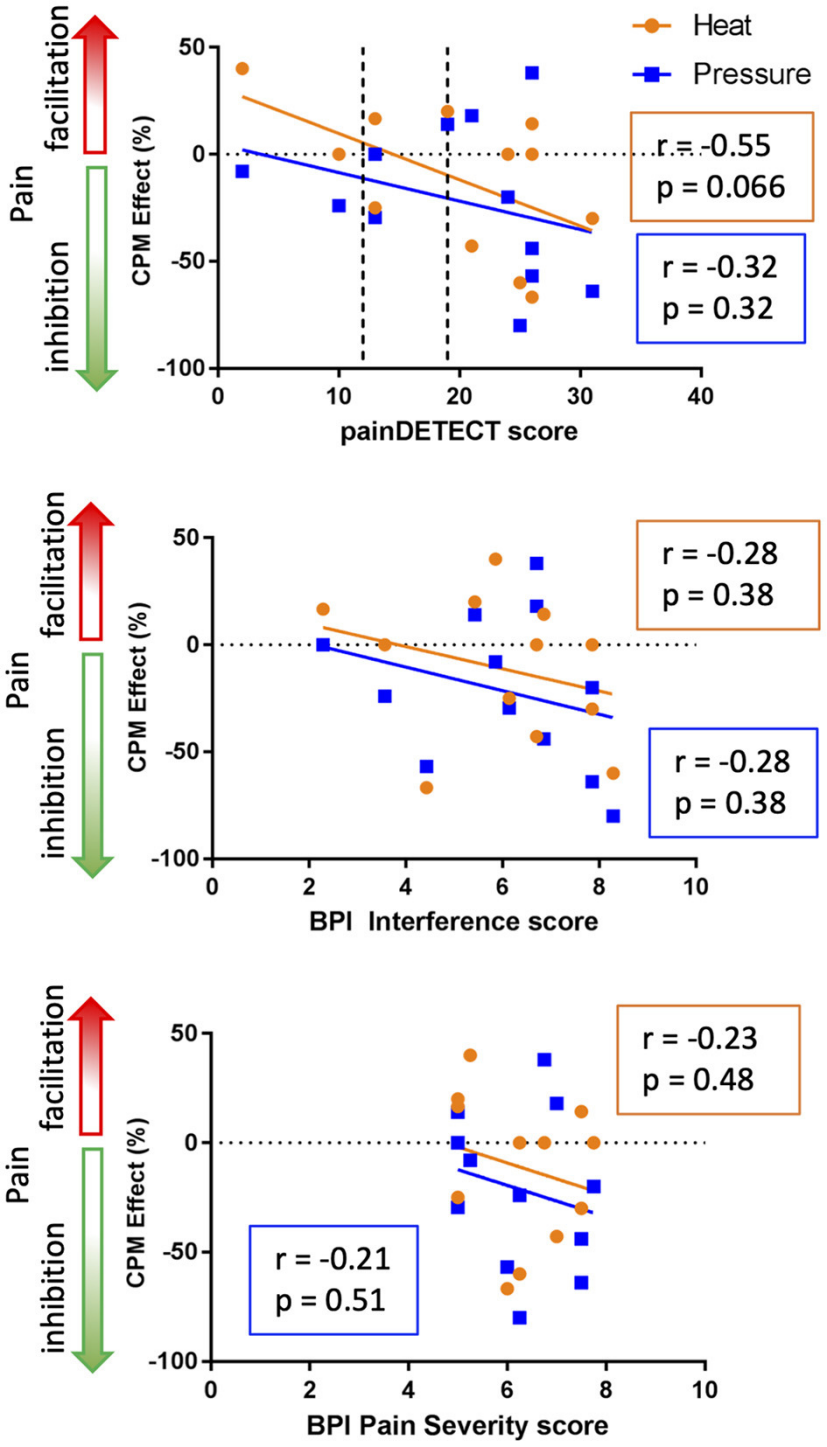
Relationship between clinical pain and CPM. The top graph indicates that there is a trend toward greater inhibitory heat-based CPM effects in those people with higher levels of neuropathic pain based on PainDetect (*r* = −0.55, *p* = 0.066). In the painDETECT questionnaire scores 12 or lower to indicate non-neuropathic pain, 13–18 mixed neuropathic pain, and 19 or greater indicates the presence of neuropathic pain is likely (categories indicated by the dotted lines). The middle and bottom graphs depict the relationship between CPM and the Brief Pain Inventory (BPI) interference and pain severity scores. The BPI interference and pain severity scores are negatively correlated with CPM effect but neither of these correlations are significant (*p* < 0.05).

The relationships between CPM and measures of clinical pain (i.e., painDETECT, BPI pain interference, and BPI pain severity) are shown in Figure 5. In general, both the heat-based (*r* = −0.55), and pressure-based (*r* = −0.32) CPM effects were not significantly correlated with painDETECT scores (heat: *t* = −2.06, *p* = 0.066; pressure: *t* = −1.10, *p* = 0.32). In addition, the heat-based CPM (*r* = −0.28) and pressure-based CPM (*r* = −0.28) were not significantly correlated with BPI average interference scores (heat: *t* = −0.93, *p* = 0.38; pressure: *t* = −0.92, *p* = 0.38). Similarly, the heat-based CPM (*r* = −0.23) and pressure-based CPM (*r* = −0.21) were not significantly correlated with BPI average pain severity scores (heat: *t* = −0.73, *p* = 0.48; pressure: *t* = −0.68, *p* = 0.51). Overall, all the clinical pain parameters showed non-significant trends toward being negatively correlated with both CPM paradigms, with the heat-based CPM and painDETECT scores showing the strongest correlation that approached significance (*r* = −0.55, *p* = 0.066).

## Discussion

This study sought to interrogate paradigms that could potentially be used to evaluate CPM consistently and easily for research and clinical investigations. Toward this goal, our study aim was to compare heat and pressure paradigms that can be used to evaluate CPM in healthy individuals and those with chronic pain. Our main findings were that 1) in healthy individuals, the pressure-based paradigm produced a stronger inhibitory CPM compared to the heat-based paradigm, and most participants exhibited modality-dependent CPM effects (i.e., inhibitory vs. facilitatory), 2) in people with chronic pain, there was no significant difference in the CPM evoked by the pressure- and heat-based paradigms, with the majority exhibiting modality-independent CPM effects, 3) the pressure paradigm evoked a similar (in chronic pain individuals) or more inhibitory CPM effect (in healthy individuals) when calculating CPM based on the Pain50 level compared to the pain detection threshold (PDT), 4) The healthy group had a significantly more inhibitory CPM than the chronic pain group in the pressure paradigm but not the heat paradigm. Overall, our findings indicate that the interpretation of CPM effects in healthy individuals needs to consider the stimulus modality and metric used to calculate CPM. Importantly though, given that the heat and pressure CPM paradigms evoke similar CPM magnitudes in chronic pain, our findings provide support for the adoption of a faster, more robust and more easily administered pressure-based paradigm to assess CPM in clinical populations and in research environments.

Overall, across subjects, the average pain evoked by a pressure stimulus induced a more inhibitory CPM effect compared to pain evoked by a heat stimulus. Possible factors underlying this finding could include that the pressure stimulus can recruit A-beta fibers and the pressure cuffs can induce additional recruitment of nociceptors from the deep-somatic stimulus and from the larger surface area compared to thermodes and this could have triggered a greater response of the descending anti-nociceptive control system (26–28). Curiously though, we found that it was only in the healthy individuals that the pressure paradigm induced a significantly more robust inhibitory CPM effect than the heat paradigm, whereas there was no significant difference between the CPM effect induced by these paradigms in individuals with chronic pain. This suggests that the assumption that a similar CPM effect can be achieved by any stimulus modality holds true for those with chronic pain but not healthy individuals.

A large systematic review of CPM studies highlighted that regardless of stimulus modality (electrical, mechanical, etc.) the majority of studies did not find any significant correlations between CPM and clinical attributes and manifestations of pain (e.g., pain intensity, severity disability, interference, and duration) (29). This aligns with our findings that BPI and PainDetect scores did not significantly correlate with CPM from the pressure paradigm. Interestingly though, this systematic review noted an exception to their overall conclusion was for the studies that used thermal TS and CS, 55% of these studies had negative significant correlations between CPM and clinical pain (29). They also found across all the studies that the clinical manifestations of pain and the CPM effect were not significantly correlated in any of the studies in idiopathic pain and most of the studies with nociceptive pain, although, about half of the studies of neuropathic pain did report a significant correlation between CPM and pain (29). Taken together these findings support our finding that the strongest correlation of heat-based CPM with a clinical pain measure was with the painDETECT scores (an assessment of neuropathic pain). A link between neuropathic pain and CPM particularly in heat-based paradigm but not a pressure paradigm, was also recently found in a study of CPM effects using heat and pressure test paradigms in people with painful and non-painful diabetic polyneuropathy (DPN) (30). This study reported that the inhibitory CPM evoked by heat was significantly correlated with greater neuropathic pain (30). This suggests that in our population of people with chronic pain, the severity of their clinical pain and its interference on activities of daily living may not greatly impact their ability to exhibit an experimentally-induced CPM. However, the neuropathic nature of their pain may impact the heat-based CPM, but not the pressure-based CPM. This finding suggests that the experimental pressure-based CPM test can be used to assess CPM ability in patients with chronic pain regardless of the severity, neuropathic aspect, and interference of their chronic pain.

Despite CPM not necessarily being related to the magnitude of clinical pain, it nonetheless holds great potential clinical utility because a weaker inhibitory CPM in individuals with chronic pain can distinguish them from healthy individuals with stronger inhibitory CPM (13, 29). This is thought to be due to individuals with weak inhibitory CPM being more at risk to develop chronic pain and/or once chronic pain develops it exhausts the pain inhibition capacity leading a weaker inhibitory CPM than healthy individuals (2). This aligns with our findings in the pressure paradigm where the healthy group had a significantly more inhibitory CPM than the chronic pain group, however this was not the case in the heat-paradigm. Clinical translation of a CPM paradigm could guide treatment selection for individuals in that it could assess the risk of post-operative chronic pain and predict treatment efficacy (4). Since CPM in the chronic pain individuals was not significantly different between pressure and heat-based paradigms, either could be translated to the clinics. The main limitation of translating the pressure cuff-based CPM paradigm is that it cannot be used on those with cardiovascular health concerns. However, the limitation of the heat paradigm is that despite the familiarization and habituation step used to find Pain50 in the heat paradigm, often the first TS1 is still not rated at a pain intensity of 50/100 and therefore extra time was then needed to re-calibrate the Pain50 before running the CPM protocol. In the pressure paradigm this was never necessary because the pain tolerance threshold could be reached in under 100s, we often could run the paradigm in under 10 min. An additional practical challenge with the heat paradigm is that care had to be taken so that the thermode lays flat on the skin, but the straps are not too tight to avoid pressure confounding the pain intensity rating of the heat stimulus. Thus, overall, the pressure paradigm was faster to run, simpler to administer, and the equipment is somewhat more straightforward, and thus may be more adaptable to a clinical setting.

One limitation of comparing a heat to a pressure paradigm is that elements of the protocols were different for the two approaches which limits an exact head-to-head comparison. For example, the heat paradigm was based on the administering rapidly increasing intensity of stimuli for blocks of time and asking a participant to provide a single rating of the evoked pain intensity. In contrast, in the pressure paradigm, stimuli were delivered continuously at a slowly increasing intensity and participants provided continuous ratings of pain intensity. However, it is of note that in the pressure protocol, we calculated CPM using the change in pain rated at Pain50 rather than the change in pressure pain detection thresholds that has been used by some research labs (21–23). We chose this approach so that we could more directly compare the pressure paradigm to heat paradigm which used Pain50 to avoid floor effects (20, 31). Additionally, much of what is known of the underlying mechanisms of CPM are based on animal studies of DNIC that used suprathreshold stimuli, and this is similar to using the suprathreshold metric of Pain50 stimuli. In contrast a determination of CPM as a threshold change (i.e., PDT) may be due to a different underlying mechanism. For example, a change in pain evoked by a suprathreshold stimulus may be more reflective of a mechanism related to hyperalgesia rather than a measure of a threshold change which may be more akin to an allodynia mechanism. Although assessing pressure CPM at Pain50 is a departure from the approach used by other labs, we note that in individuals with chronic pain there was no significant difference in pressure CPM calculated using Pain50 vs. PDT. However, in healthy individuals using Pain50 resulted in a significantly stronger inhibitory CPM compared to using PDT. Unexpectantly, the mean Pain50 pressure was higher in the chronic pain group than in the healthy control group. The reason for this is not clear given that the PDT pressures were similar in the healthy and chronic pain groups, suggesting that sensory loss is not a factor. However, healthy individuals unfamiliar with deep intense pain may be more sensitive to the pressure stimulus and thus have a lower Pain50 pressure compared to those with chronic pain whose ratings to the stimulus may be perceived as relatively less painful given their experience from their condition.

The relatively small sample size and diversity in types of chronic pain conditions in this study may have precluded the ability to detect significant correlations between clinical measures and CPM in the chronic pain group. This sample size limitation also did not allow us to carry out an extensive sex and age effect analysis in either group. Although the proportion of males and females in our study was not significantly different between groups, the healthy group was significantly younger than the chronic pain group. Interestingly, a recent study in healthy individuals found the age effect was larger than the sex effect; across all the different stimulus modality paradigms younger males had the strongest inhibitory CPM (32). This aligns with previous findings showing that younger and healthy individuals on average have a stronger inhibitory CPM than older individuals and those with chronic pain (13). Here in our study as well, the somewhat younger healthy group had a significantly more inhibitory CPM than the older chronic pain group in the pressure paradigm. Therefore, the CPM we found to be evoked at Pain50 in the pressure paradigm was more consistent with age and health effects in the literature than the heat paradigm.

In conclusion, this study found that a pressure-based CPM paradigm evoked an inhibitory CPM effect in most participants (healthy individuals and those with chronic pain), but was significantly stronger than the heat-based CPM only in the healthy individuals. Similarly, CPM in the pressure-based CPM paradigm at Pain50 was significantly more inhibitory than at pressure pain detection threshold in healthy individuals. In individuals with chronic pain the paradigm type is not critical because the pressure CPM does not significantly differ from heat CPM and PDT CPM. Given our finding that the pressure paradigm may be less impacted from clinical neuropathic pain and can be carried out using a relatively simple system with a shorter test time than heat-based paradigm, we propose that the pressure-based CPM paradigm offers a good option for use in research studies and is particularly well-suited for clinical use, potentially to aid in assessing predictive factors of treatment outcome.

## Data Availability Statement

The raw data supporting the conclusions of this article will be made available by the authors, without undue reservation.

## Ethics Statement

The studies involving human participants were reviewed and approved by University Health Network Research Ethics Board. The patients/participants provided their written informed consent to participate in this study.

## Author Contributions

KD and RE-S conceived this study. Data collected from participants with chronic pain was part of a larger study of treatment effects, clinically led by ABh. RE-S collected data with the help from CF, ABe, EM, JK, SF, and NO. All authors contributed to the drafting of the manuscript and approved the final version.

## Funding

This research was supported by the Mayday Fund, and by the Canadian Institute of Health Research (operating grant PJT-156409 to KD) and Strategy for Patient-Oriented Research (SPOR) funding of the Canadian Chronic Pain Network (SCA-145102).

## Conflict of Interest

The authors declare that the research was conducted in the absence of any commercial or financial relationships that could be construed as a potential conflict of interest.

## Publisher's Note

All claims expressed in this article are solely those of the authors and do not necessarily represent those of their affiliated organizations, or those of the publisher, the editors and the reviewers. Any product that may be evaluated in this article, or claim that may be made by its manufacturer, is not guaranteed or endorsed by the publisher.
